# Translating umbilical cord blood extracellular vesicles into a novel therapy for postpartum alopecia: a mechanistic and preclinical review

**DOI:** 10.3389/fimmu.2026.1872590

**Published:** 2026-09-25

**Authors:** Yi Yang, Min Li, Haiwei Chen, Huanzhi Jin, Yue Lin, Xueliang Zhang, Zheyan Chen

**Affiliations:** 1 Clinical Laboratory, Wenzhou People’s Hospital, Wenzhou, China; 2 Wenzhou Third Clinical Institute Affiliated to Wenzhou Medical University, Wenzhou, China; 3 The Third Affiliated Hospital of Shanghai University, Wenzhou, China; 4 The Wenzhou People’s Hospital Affiliated of Hangzhou Medical College, Wenzhou, China; 5 Department of General Practice, Wenzhou People’s Hospital, Wenzhou, China; 6 Department of Plastic and Reconstructive Surgery, Wenzhou People’s Hospital, Wenzhou, China

**Keywords:** extracellular vesicles, hair follicle regeneration, molecular mechanisms, postpartum alopecia, therapeutic efficacy, umbilical cord blood

## Abstract

Postpartum alopecia (PPA) is a common physiological hair loss in women, characterized by complex pathogenesis involving abrupt postpartum hormonal withdrawal, disrupted hair follicle cycling, local microenvironmental inflammation, and oxidative stress. Umbilical cord blood-derived extracellular vesicles (UCB-EVs)—nanosized, lipid-bilayer-enclosed particles enriched with microRNAs, growth factors, and proteins—have shown considerable promise in regenerative medicine owing to their low immunogenicity, high biocompatibility, and tissue-repair capacity. This review systematically summarizes the current state of research on UCB-EVs in the context of PPA, focusing on their proposed molecular mechanisms in hair follicle regeneration, hair-cycle modulation, antiinflammatory effects, and antioxidative stress responses. Importantly, the available evidence remains largely preclinical, derived from *in vitro* and animal-model studies; direct clinical evidence for UCBEVs in PPA is still lacking. UCB-EVs should therefore be regarded as a promising investigational strategy, and their clinical translation will require rigorous validation of standardization, safety, and efficacy.

## 1 Introduction

Female postpartum alopecia (PPA) represents a significant clinical and psychosocial challenge, emerging as one of the most common dermatological conditions affecting women in the postpartum period. Clinically, it is characterized by diffuse hair shedding that typically manifests three to six months following childbirth, reflecting a synchronized shift of a large proportion of hair follicles from the active anagen phase into the resting telogen phase (1). This phenomenon, termed telogen effluvium (TE), is widely recognized as the primary driver of postpartum hair loss (1). The pathophysiological foundation of PPA is deeply rooted in the dramatic endocrine changes that occur after delivery. The abrupt and substantial decline in circulating estrogen and progesterone levels removes the pro-anagenic and anti-apoptotic signals that these hormones provide during pregnancy, thereby triggering a cascade of events that disrupt the normal hair cycle. Beyond mere hormonal withdrawal, the local follicular microenvironment experiences a state of heightened inflammation, with elevated levels of proinflammatory cytokines such as interleukin-6 (IL-6) and tumor necrosis factor-alpha (TNF-α) (2). This inflammatory milieu is further compounded by increased oxidative stress, which damages the cellular components of the hair follicle, and a relative insufficiency in perifollicular angiogenesis, which compromises the nutrient and oxygen supply necessary for robust hair shaft production (2). The resulting condition is not merely a cosmetic concern; the psychological impact of significant hair loss can be profound, leading to heightened anxiety, diminished self-esteem, and a substantial decline in quality of life. Epidemiological data indicates that the prevalence of PPA is remarkably high, with studies suggesting that over 90% of postpartum women experience some degree of hair loss (3). Furthermore, the problem is often more complex than a simple, isolated TE. Clinical investigations have demonstrated that postpartum TE frequently unmasks or exacerbates pre-existing, subclinical hair loss disorders, such as androgenetic alopecia (AGA) and traction alopecia (TA) (4, 5). Dermoscopic evaluations in cohorts of women with postpartum hair loss have revealed that only a minority present with pure TE, while the majority exhibit a combination of TE with AGA, TA, or both (5). This overlap underscores the necessity for a nuanced diagnostic approach, as the treatment strategies for each underlying condition differ significantly. The identification of these latent disorders is critical for appropriate and effective management, moving beyond symptomatic treatment to address the specific root causes of hair loss in each individual (1, 5).

The current therapeutic landscape for PPA is remarkably limited, often characterized by modest efficacy, slow onset of action, and potential side effects, highlighting a significant unmet clinical need. The most commonly recommended first-line intervention is topical minoxidil, a vasodilator that prolongs the anagen phase and stimulates hair follicle growth. However, its efficacy in the acute, self-limited setting of PPA is debated, and clinical responses vary significantly. Furthermore, its use is associated with side effects such as scalp irritation and hypertrichosis, and it necessitates continuous application to maintain results. Low-level laser therapy (LLLT) offers a non-pharmacological alternative, proposed to enhance cellular metabolism and blood flow within the scalp, yet the evidence base supporting its use specifically for PPA remains limited. Nutritional supplementation with vitamins and minerals such as iron, zinc, and biotin is a common adjunct, but its impact is primarily supportive and unlikely to reverse the primary hormonal and inflammatory drivers of the condition. The psychological implications of PPA are significant. A recent cross-sectional study has established a significant, independent association between the severity of postpartum hair loss and maternal anxiety, as measured by the Generalized Anxiety Disorder 2-item scale (GAD-2) (6). Women reporting very significant hair loss were over four times more likely to experience clinically relevant anxiety compared to those with no hair loss (6). This finding underscores that PPA is not a trivial condition but a genuine health concern that can negatively impact maternal mental health, bonding with the infant, and overall family well-being. Factors such as long-term breastfeeding and a history of preterm labor have been identified as independent predictors of more pronounced postpartum hair loss, further complicating the clinical picture (3). Given these limitations and the high prevalence of the condition, there is a pressing and urgent need for novel, more effective, and targeted therapeutic strategies that can address the complex pathophysiology of PPA, including its inflammatory, oxidative, and angiogenic components, while offering a favorable safety profile.

In this context, UCB-EVs have emerged as particularly promising and innovative therapeutic candidates, leveraging a readily available, non-invasive, and ethically sound biological resource. Umbilical cord blood, a byproduct of childbirth typically discarded, represents a rich source of multipotent mesenchymal stem cells (MSCs) and a wide array of bioactive molecules. The secretome of these MSCs, particularly their extracellular vesicles, has attracted significant attention in regenerative medicine. Extracellular vesicles, including exosomes and microvesicles, are nanoscale, lipid-bilayer-enclosed particles naturally secreted by virtually all cell types. They function as vital mediators of intercellular communication by shuttling a complex cargo of proteins, lipids, and nucleic acids—most notably microRNAs (miRNAs), messenger RNAs (mRNAs), and other non-coding RNAs—from their parent cell to recipient cells (2, 7). This delivery system allows UCB-EVs to exert profound paracrine effects without the risks associated with direct cell transplantation, such as immune rejection, tumorigenicity, and ectopic tissue formation. The therapeutic potential of MSC-derived EVs in dermatology and hair biology is rapidly accumulating (7). Preclinical studies have demonstrated that exosomes derived from human umbilical cord MSCs (hUCMSCs) can significantly promote hair follicle regeneration and growth in models of androgenetic alopecia (2). These EVs have been shown to rescue hair follicles from dihydrotestosterone (DHT)-induced damage, enlarge hair follicle size, and facilitate the transition of resting telogen follicles back into active anagen follicles (2). The mechanisms underpinning these effects are multifaceted and directly target the core pathological processes implicated in PPA. At the molecular level, UCB-EVs are enriched with specific miRNAs, such as miR-21-5p and let-7b-5p, which are capable of targeting and modulating key signaling pathways critical for hair follicle biology (2). Notably, these miRNAs activate the canonical Wnt/β-catenin signaling cascade, a master regulator of hair follicle morphogenesis, cycling, and stem cell activation. By upregulating Wnt/β-catenin signaling, UCB-EVs stimulate the proliferation and functional differentiation of dermal papilla cells (DPCs), which are the key signaling centers of the hair follicle, thereby promoting robust hair shaft formation (2). Given this compelling background, the application of UCB-EVs to the specific context of PPA presents a logical and exciting frontier. The biological activities of UCB-EVs—including their potent pro-angiogenic, anti-inflammatory, anti-apoptotic, and immunomodulatory properties—are ideally suited to counteract the multiple pathogenic insults that characterize the PPA hair follicle. While the role of UCB-EVs in addressing androgenetic alopecia is supported by dedicated *in vitro* and *in vivo* preclinical data (2), their potential to mitigate the acute, diffuse telogen effluvium seen postpartum, which is driven by distinct hormonal and inflammatory triggers, has not been systematically explored. This review aims to bridge this critical knowledge gap. We will first establish a comprehensive understanding of the pathophysiology of PPA, integrating the latest clinical evidence on its epidemiology, psychological impact, and underlying mechanisms. Subsequently, we will delve into the molecular biology of UCB-EVs, detailing their biogenesis, cargo profiles (particularly miRNAs), and mechanisms of action in promoting hair growth. We will then separate two tiers of pre-clinical evidence: (1) direct *in vitro* hormone-induced telogen arrest and postpartum mouse model data specific to PPA; (2) indirect correlative findings extrapolated from AGA, wound healing, and inflammatory skin disease models, with explicit notation where mechanistic inference relies solely on non-PPA experimental systems. Finally, this review critically evaluates the clinical translation challenges associated with this approach, including standardization of EV isolation, characterization, and delivery methods, as well as considerations for safety and large-scale production. By systematically reviewing the current literature, this review provides a foundational framework for future research and the development of novel, regenerative therapies for the millions of women affected by postpartum hair loss. Although the convergence of an easily accessible, cell-free biological therapy with a clear unmet clinical need makes UCB-EVs an attractive candidate, it must be emphasized that the current evidence is largely preclinical. UCB-EVs should therefore be positioned as a promising investigational approach rather than an established therapy; whether they can move beyond symptomatic relief toward true therapeutic regeneration awaits rigorous clinical validation.

## 2 Biological characteristics and preparation of UCB-EVs

### 2.1 Definition and classification of UCB-EVs

Extracellular vesicles (EVs) are lipid bilayer-enclosed structures released by cells, playing crucial roles in intercellular communication by transporting bioactive molecules such as proteins, lipids, and RNAs (8). They are broadly classified into exosomes (30–150 nm), microvesicles (100–1000 nm), and apoptotic bodies (>1000 nm) based on their biogenesis and size (9). It is important to clarify that apoptotic vesicles represent a heterogeneous population of vesicles released during programmed cell death, whereas apoptotic bodies (ApoBDs) specifically refer to the larger membrane-wrapped fragments (typically 1–5μm in diameter) generated through apoptotic cell disassembly, often containing nuclear fragments, chromatin remnants, and organelle contents (10). Notably, emerging evidence has revealed that ApoVs possess significant therapeutic and regenerative potential beyond their traditional role as cellular debris (11). Recent studies have demonstrated that mesenchymal stem cell-derived apoptotic vesicles (MSC-ApoVs) can promote angiogenesis and tissue regeneration in models of hind limb ischemia through the NAMPT/SIRT1/FOXO1 axis (11). UCB is a rich source of naïve cells with strong regenerative potential, and the EVs derived from it primarily originate from resident MSCs, hematopoietic stem cells, and endothelial progenitor cells (8, 12). Exosomes are of particular interest due to their cargo of regulatory molecules. For instance, UCB mononuclear cell-derived sEVs (UCB-MNC-sEVs, a subset of total UCB-EVs) are enriched with specific microRNAs (miRNAs) like miR-150, which possess antiinflammatory and regenerative capacities (13). These EVs also carry growth factors such as vascular endothelial growth factor (VEGF) and hepatocyte growth factor (HGF), which are implicated in promoting angiogenesis and tissue repair (14). All UCB-derived vesicle subtypes (total UCB-EVs, UCB-MSC-EVs, isolated exosomes) expresses characteristic tetraspanin proteins like CD9, CD63, and CD81, which serve as identification markers and are commonly detected via Western blot analysis (13, 15). Their internal payload is complex and includes key molecules for regulating biological processes; for example, they contain factors that can activate the Wnt/β-catenin pathway and anti-inflammatory cytokines that modulate immune responses (8, 16). When compared to EVs derived from adult tissues, UCB-EVs exhibit lower immunogenicity and greater potential to promote cell proliferation and regeneration (17, 18). This advantageous profile is attributed to the ontogenetically early developmental stage of cord blood cells, resulting in an EV miRNA repertoire that is skewed toward supporting cell proliferation, tissue regeneration, and immunomodulation rather than promoting inflammatory or senescent pathways (8, 19). This fundamental biological distinction underpins the therapeutic interest in UCB-EVs for regenerative applications, including those targeting conditions like postpartum alopecia. In this review, the term UCB-EVs refers to the total EV population isolated from umbilical cord blood or from the conditioned medium of cord blood-derived mononuclear cells, and includes exosomes, microvesicles, and other subtypes. UCBMSC-EVs specifically denotes EVs harvested from culture supernatants of expanded and characterized umbilical cord blood mesenchymal stem cells. Exosomes (or small EVs, sEVs) refers to the 30–150 nm subpopulation enriched in tetraspanins (CD9, CD63, CD81). Because these preparations differ in source, isolation method, size range, and marker profile, they cannot be used interchangeably. Throughout this review, we retain the original nomenclature used in each cited study and specify the isolation method and characterization data were available, in line with MISEV guidelines (17).

### 2.2 Isolation and purification techniques for UCB-EVs

The isolation and purification of UCB-EVs are critical steps that directly impact their purity, functionality, and suitability for therapeutic applications. Commonly employed methods include ultracentrifugation (UC), size-exclusion chromatography (SEC), polyethylene glycol (PEG) precipitation, and immunoaffinity capture (13, 20). Ultracentrifugation has long been considered the gold standard but is time-consuming, may compromise EV structural integrity due to high g-forces, and can co-precipitate protein aggregates (13). In contrast, techniques like tangential flow filtration combined with SEC (UF/SEC) offer significant advantages in terms of production time, standardization, scalability, and vesicle yield. One optimized protocol using UF/SEC recovered approximately 400 times more sEVs per milliliter of media compared to UC and allowed for upscaling that further increased yield by about threefold (13). This method effectively minimizes downstream contaminants such as lipoproteins and protein aggregates, which is crucial for obtaining a high-purity (>90%) and homogenous EV population (13). Immunoaffinity methods, which utilize antibodies against specific surface markers (e.g., CD63, CD81), can enrich for particular EV subpopulations but are often associated with higher costs and may not be suitable for large-scale production (20). Rigorous quality control is essential to ensure batch-to-batch consistency, especially for clinical translation. This typically involves a combination of techniques: This typically involves a combination of techniques: dynamic light scattering (DLS), also known as photon correlation spectroscopy, which measures time-dependent fluctuations in scattered light caused by Brownian motion of particles to determine the hydrodynamic diameter and polydispersity index (PDI) of EVs (21); Nanoparticle tracking analysis (NTA) to determine particle size distribution and concentration (13, 14); Western blot analysis to confirm the presence of EV protein markers such as CD63, CD81, and TSG101 (13, 15); and transmission electron microscopy (TEM) or cryo-electron microscopy (cryo-EM) to verify the characteristic spherical, lipid bilayer morphology of the vesicles (14, 22). Establishing such standardized preparation and characterization pipelines is paramount for preclinical research to ensure the reliability and reproducibility of studies investigating UCB-EVs’ therapeutic potential (13).

### 2.3 Biological cargo and function of UCB-EVs

The therapeutic potential of UCB-EVs is largely attributed to their diverse and bioactive molecular cargo, which includes microRNAs (miRNAs), proteins, and lipids. RNA sequencing and miRNA profiling of UCB-EVs reveal a rich content of miRNAs involved in regulating critical cellular processes. For instance, UCB mononuclear cell-derived sEVs (UCB-MNCsEVs) are enriched with miR-150, which is associated with anti-inflammatory and regenerative functions (13). Other studies on EVs from different perinatal sources highlight miRNAs like miR-124-3p, which has demonstrated neuroprotective and pro-neurogenic effects when delivered via UCB-MNC-sEVs in a Parkinson’s disease model (23). These miRNAs can modulate key signaling pathways; for example, miR-101 encapsulated in EVs from human umbilical cord mesenchymal stem cells (HUCMSCs) was shown to promote trophoblast proliferation and migration by targeting the BRD4/NF-κB/CXCL11 axis, a mechanism relevant to tissue repair (24). Similarly, EV-derived miR-18b was found to enhance trophoblast function by downregulating the Notch2/TIM3/mTORC1 pathway (25). Proteomic analyses further demonstrate that UCB-EVs carry a multitude of functional proteins. They are laden with growth factors such as VEGF, HGF, insulin-like growth factor-1 (IGF-1), and platelet-derived growth factor (PDGF), which are instrumental in stimulating angiogenesis and improving nutrient supply to tissues, a process vital for hair follicle regeneration (14). Additionally, UCB-EVs contain potent anti-inflammatory proteins like interleukin-10 (IL-10) and tissue inhibitor of metalloproteinases-1 (TIMP-1) (8). For example, UCB-MNC-sEVs polarize macrophages toward an anti-inflammatory phenotype, reduce T-cell proliferation, and support regulatory T-cell development, thereby alleviating inflammatory conditions (8). Importantly, the immunomodulatory and regenerative properties of UCB-EVs extend significantly to dermatological applications, particularly in inflammatory skin diseases and skin rejuvenation therapies. Recent studies have demonstrated that UCB-MSC-derived EVs exhibit remarkable therapeutic efficacy in inflammatory skin conditions such as psoriasis and atopic dermatitis (AD). In a 3D model of psoriatic epidermis, UCB-MNC-sEVs significantly decreased the expression of antimicrobial peptides S100A7 and DEFB4, as well as proinflammatory mediators IL-6, IL-8, CXCL10, and COX-2 (26). Furthermore, *in vivo* studies showed that these sEVs significantly prevented or reversed epidermal thickening (acanthosis) in an imiquimod-induced psoriasis model, while tendentially increasing the number of regulatory T-cells (Treg) in affected skin (25). Similarly, in atopic dermatitis models, UCMSC-EVs-enhanced keratinocyte proliferation, restored epidermal structural proteins, and reduced inflammatory mediators, thereby improving skin barrier function and reducing AD severity (26). These findings underscore the capacity of UCB-EVs to simultaneously address immune dysregulation and barrier defects in inflammatory skin diseases, positioning them as compelling candidates for long-term dermatological therapy (26, 27).

Beyond inflammatory conditions, UCB-EVs have emerged as promising agents for skin rejuvenation and aesthetic dermatology. Human umbilical cord blood mesenchymal stem cell-derived exosomes (UCB-MSC-Exos) contain high concentrations of growth factors such as epidermal growth factor (EGF), which have been shown to increase collagen production and migration ability of normal fibroblasts (28). These exosomes penetrate into the epidermis of skin samples in a time-dependent manner and increase collagen I and elastin expression while decreasing matrix metalloproteinase-1 (MMP1) expression, thereby counteracting extracellular matrix degradation (28). Additionally, UC-MSC-EVs promote dermal fibroblast proliferation and matrix metalloproteinase expression while suppressing melanin synthesis in melanocytes, highlighting their multifaceted potential in skin rejuvenation, scar reduction, and pigmentation control (27). The cosmetic and therapeutic benefits of UCB-EVs for skin care are mediated through their immunomodulatory function, reduction of oxidative stress, decreasing senescence, and stimulation of extracellular matrix (ECM) components production (28). These dermatological applications—encompassing both inflammatory skin disease management and skin rejuvenation—are directly relevant to conditions such as postpartum alopecia, where scalp inflammation and compromised skin barrier function may contribute to hair follicle dysfunction.

The lipid composition of the EV membrane, including sphingomyelin and cholesterol, is not only crucial for membrane stability and integrity but also facilitates membrane fusion with target cells and participates in signal transduction (13). This composite cargo—encompassing regulatory miRNAs, mitogenic and angiogenic proteins, and immunomodulatory factors— forms the integrated molecular foundation through which UCB-EVs can orchestrate processes such as reducing inflammation, promoting vascularization, and stimulating cellular proliferation and differentiation, which are all essential for fostering a regenerative microenvironment conducive to hair follicle recovery.

## 3 Pathogenesis of postpartum hair loss in women and limitations of current treatments

### 3.1 Hormonal regulation mechanisms of postpartum hair loss

Distinction of Postpartum Telogen Effluvium (PP-TE) from other alopecias: PP-TE is a transient, diffuse, global follicle cycle synchronization disorder triggered exclusively by peripartum estrogen/progesterone collapse, reversible with hormonal recovery; this differs fundamentally from androgenetic alopecia (AGA, progressive follicle miniaturization driven by DHT, frontal/vertex-predominant thinning) and traction alopecia (TA, focal scarring/nonscarring loss from mechanical tension, temporal/marginal predilection). Most PP patients present mixed PP-TE + subclinical AGA/TA, but core PP pathology relies on synchronized mass telogen entry rather than permanent follicle atrophy. The pathogenesis of postpartum telogen effluvium is primarily driven by the dramatic hormonal shifts that occur following childbirth, which significantly disrupts the normal hair growth cycle. During pregnancy, a sustained elevation in estrogen levels significantly prolongs the anagen (growth) phase of hair follicles, inhibiting the physiological transition to the catagen and telogen phases. This hormonal milieu induces a large proportion of follicles to remain synchronized in anagen, accounting for the increased hair density and thickness often observed in pregnant women. The postpartum period is marked by a precipitous decline in estrogen, which withdraws this protective, growth-sustaining signal. Consequently, a massive and synchronous shift of hair follicles from the anagen phase into the telogen (resting) phase is triggered, typically occurring 2 to 4 months after delivery. This synchronized entry into telogen is the hallmark physiological mechanism leading to the diffuse hair shedding known as telogen effluvium, commonly presenting around 3 to 6 months postpartum (29). The abrupt withdrawal of estrogen is the primary driver of this cycle disruption.

In addition to estrogen, other hormonal fluctuations play significant contributory roles in postpartum hair loss. The progesterone level, which also rises during pregnancy, falls sharply after delivery. While progesterone’s direct effects on the hair follicle are complex, its decline may indirectly exacerbate hair loss by influencing the expression of androgen receptors. This alteration can potentially increase the follicle’s sensitivity to circulating androgens, thereby compounding the hair-shedding process (29). Furthermore, elevated prolactin levels, necessary for lactation, can directly inhibit hair follicle stem cell activity. High prolactin levels are known to suppress follicular function and delay the recovery of the normal hair cycle, thus prolonging the duration of the telogen effluvium episode (29). This multi-hormonal interplay means postpartum hair loss is not simply a consequence of estrogen withdrawal but a complex condition driven by the simultaneous imbalance of estrogen, progesterone, and prolactin.

The role of thyroid function is another critical, yet often underdiagnosed, hormonal factor in postpartum hair loss. The postpartum period is a high-risk time for the development of thyroid dysfunction, most notably postpartum thyroiditis, which can manifest as either transient hyperthyroidism, hypothyroidism, or both (30). Thyroid hormones are essential for the proper regulation of the hair follicle cycle, including keratinocyte proliferation and differentiation. Both hyperthyroidism and hypothyroidism are well-established causes of diffuse hair loss, including telogen effluvium and other non-scarring alopecias (31). The mechanism involves the disruption of thyroid hormone receptor signaling within the dermal papilla and outer root sheath cells, which impairs normal hair shaft production and cycle progression. In fact, the prevalence of thyroid abnormalities in patients with telogen effluvium has been documented to be between 5.7% and 17%, underscoring the importance of screening for postpartum thyroiditis in patients presenting with persistent postpartum hair loss (30). This connection is so significant that topical thyroid hormone therapy, such as L-thyroxine, has been proposed as a potential therapeutic avenue for telogen effluvium, given its well-documented impact on human hair follicle physiology (32).

Finally, the overall hormonal environment must be recognized as a mosaic of interacting endocrine pathways. For example, the hypothalamic-pituitary-adrenal (HPA) axis, activated by the stress of childbirth and the postpartum period, leads to elevated cortisol levels. Stress-induced cortisol can directly contribute to hair follicle cycle disruption, pushing follicles into telogen (29). Furthermore, nutritional deficiencies that are common in the postpartum period, such as low serum ferritin and vitamin D, are intimately linked to the hormonal changes and exacerbate hair shedding. Studies show that low ferritin is significantly associated with telogen effluvium, and vitamin D, which acts as a hormone, is crucial for proper hair follicle cycling through its involvement in signaling pathways of growth and differentiation (33–35). Therefore, the hormonal control of postpartum hair loss is a multifactorial system where the rapid decline of estrogen, imbalanced progesterone and prolactin, potential thyroid dysfunction, and stress-induced cortisol activity combine to create a perfect storm that disrupts hair follicle homeostasis and precipitates telogen effluvium.

From a biological standpoint, PPA is fundamentally an acute, diffuse telogen effluvium triggered by the postpartum decline in estrogen and progesterone, and is typically self-limited with no permanent destruction of follicle structure. This distinguishes it from androgenetic alopecia (AGA), which is a chronic, progressive miniaturization driven by DHT-androgen receptor signaling; from traction alopecia (TA), which results from mechanical damage; and from alopecia areata (AA), which is an autoimmune, T-cell-mediated attack on the follicle. Consequently, findings from AGA or AA models cannot be directly extrapolated to PPA. In clinical practice, PPA may also overlap with AGA, TA, or both—indeed, more than half of patients in some cohorts show mixed patterns—so diagnostic stratification (history, dermoscopy, laboratory testing) is required before any EV-based strategy is considered. Pathogenesis of postpartum hair loss are illustrated in **Figure 1**.

**Figure 1:**
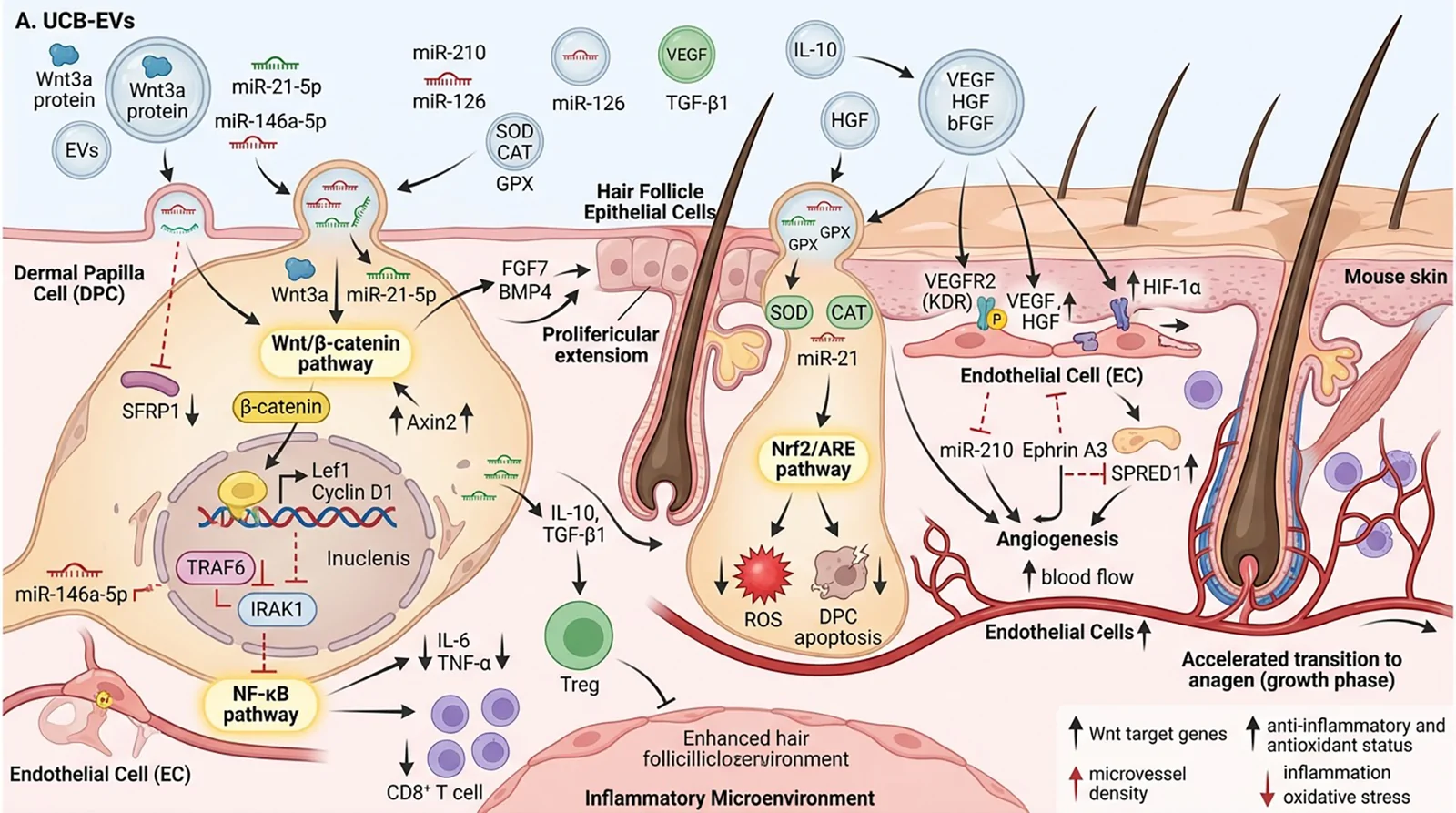
Pathogenesis of postpartum hair loss. Schematic diagram illustrating the pathophysiological cascade of postpartum hair loss. During pregnancy, high estrogen and progesterone sustain robust hair follicle growth. After delivery, rapid hormone decline initiates immune activation, pro-inflammatory cytokine release, oxidative stress, and JAK-STAT/NF-κB signaling, ultimately resulting in dermal vascular atrophy, impaired follicle nutrition, and follicle miniaturization.

### 3.2 Inflammation and oxidative stress in the hair follicle microenvironment

Beyond the initial hormonal trigger, the progression and persistence of postpartum telogen effluvium are sustained by synergistic inflammatory and oxidative stress responses within the hair follicle microenvironment. The acute physiological stress associated with childbirth and the subsequent pronounced hormonal shifts induce a systemic state of low-grade inflammation, a condition mirrored by the scalp microenvironment. Evidence from other forms of alopecia, such as that seen in COVID-19, suggests that severe systemic inflammation can dramatically increase the risk of telogen effluvium, with patients exhibiting higher levels of inflammatory biomarkers like C-reactive protein and IL-1β correlating with increased hair shedding (36).

In the context of altered immunity and inflammation, dysbiosis of the gut microbiome has been shown to impact systemic immunological balance and can contribute to increased inflammation and impaired epithelial barrier function, further influencing hair follicle health (37). This suggests that an inflammatory milieu is not merely a secondary phenomenon but a core driver of the hair follicle cycle disruption.

The recruitment of immune cells, particularly CD8+ T cells, to the perifollicular area is a hallmark feature of many types of alopecia. In non-scarring alopecias such as telogen effluvium, the observed inflammation is associated with the activation of cytokine signaling pathways. Pro-inflammatory cytokines such as interleukin-6 (IL-6), tumor necrosis factor-alpha (TNF-α), and interferon-gamma (IFN-γ) are elevated within the scalp tissue of patients with excessive hair shedding. These cytokines directly inhibit the proliferation of hair follicle stem cells and keratinocytes, by activating signaling cascades, most notably the JAK-STAT pathway (38–40). The activation of this pathway forces hair follicles to prematurely exit the anagen growth phase and enter the telogen resting phase, thereby accelerating hair shedding and preventing the initiation of a new growth cycle (41). This direct immune-mediated attack on the hair follicle is a powerful driver that distinguishes pathological hair loss from simple hormonal shedding.

Equally damaging is the role of oxidative stress, characterized by an imbalance between the production of reactive oxygen species (ROS) and the body’s antioxidant defenses. The postpartum period is a time of increased metabolic activity and physiological recovery, which paradoxically can lead to elevated ROS levels. Within the hair follicle, this oxidative environment directly targets the dermal papilla cells—a critical component that regulates hair growth. Mitochondrial dysfunction ensues, leading to dermal papilla cell apoptosis and senescence (42). Furthermore, ROS act as potent signaling molecules that activate the nuclear factor kappa B (NF-κB) pathway. NF-κB is a master transcription factor for proinflammatory genes; its activation amplifies the local production of further inflammatory cytokines, creating a self-perpetuating cycle of inflammation and oxidative damage that is detrimental to hair follicle function and survival (41). This vicious cycle is a central mechanism that can turn a single episode of telogen effluvium into a chronic or recurring condition.

The vascular supply to the hair follicle is also severely compromised in the face of these microenvironmental insults. Postpartum estrogen decline directly leads to a reduction in the production of vascular endothelial growth factor (VEGF), a key growth factor responsible for angiogenesis and maintaining the dense capillary network surrounding the hair follicle (43). The resulting microvascular damage and capillary atrophy deprive the follicle of essential oxygen and nutrients, leading to follicle miniaturization and dysfunction. Compounding this, the inflammatory cytokines and ROS damage the endothelial cells of existing blood vessels, further impairing circulation. This vascular insufficiency not only starves active hair follicles but also impairs the stem cell niche responsible for regenerating the hair follicle during the new anagen phase. Therefore, the combined effects of inflammation, oxidative stress, and vascular damage create a fundamentally hostile microenvironment for the hair follicle, transforming a hormonally-triggered event into a self-sustaining pathological state that requires targeted intervention (44).

Solid lines/boxes indicate core PPA pathophysiological pathways (hormonal withdrawal, inflammation/oxidative stress, microvascular impairment); dashed lines/boxes indicate associated or aggravating factors (AGA, TA, thyroid dysfunction, nutritional deficiency).

### 3.3 Existing treatment modalities and their limitations

Current therapeutic options for postpartum telogen effluvium are largely repurposed from treatments for other forms of alopecia and are severely limited by their mechanisms, efficacy, and safety profiles in the postpartum context. Minoxidil remains the only FDA-approved topical medication for female pattern hair loss and is frequently used off-label for telogen effluvium. It is believed to work by opening potassium channels, which increases blood flow to the hair follicle and prolongs the anagen phase (45). However, its efficacy is far from universal; approximately 30% of female patients fail to respond to treatment, and even among responders, it typically takes 3 to 6 months to observe noticeable results (46). Critically for postpartum women, there is a significant gap in safety data. No large-scale, randomized controlled trials exist to evaluate the safety of topical minoxidil during lactation, leading to great uncertainty and frequently causing clinicians to advise against its use for fear of neonatal exposure and unknown long-term effects on the infant (47).

Physical and injectable therapies, while offering alternative mechanisms, are associated with distinct limitations for women recovering from childbirth. Low-level laser therapy (LLLT) is a non-invasive modality that employs specific wavelengths of light to stimulate mitochondrial activity within hair follicle cells, thereby promoting anagen re-entry. However, the need for regular, consistent treatments spanning months, combined with the significant upfront cost of purchasing a medical-grade device, makes it a challenging regimen for new mothers who are often sleep-deprived and managing the demands of a newborn (46). Similarly, platelet-rich plasma (PRP) injections, which deliver a concentrated cocktail of growth factors to the scalp, have shown promise in stimulating follicular stem cells. Yet, this procedure is invasive, requiring multiple sessions with a dermatologist. It is associated with pain, discomfort, and risks of infection, bruising, and temporary post-injection shedding. Furthermore, PRP preparation and potency are not standardized, leading to highly variable and inconsistent outcomes between clinics and even between treatments (40). For a postpartum mother, scheduling and tolerating these regular clinical visits can be a significant barrier to consistent treatment.

Oral pharmacological agents present even greater and often insurmountable safety concerns for women of childbearing age and in the postpartum period. Medications like finasteride, a 5alpha-reductase inhibitor that blocks the conversion of testosterone to dihydrotestosterone (DHT), are absolutely contraindicated in women who are or could become pregnant due to a high risk of teratogenicity, particularly causing ambiguous genitalia in a male fetus (28). Other anti-androgens such as spironolactone, which can improve androgenic alopecia, carry potential systemic side effects, including electrolyte imbalances (hyperkalemia), menstrual irregularities, and dizziness. While sometimes used in female pattern hair loss, its safety during breastfeeding is not established, and its use is generally avoided without reliable contraception due to potential risks to a developing fetus. These risks make them unsuitable for the postpartum patient who may be considering future pregnancies or is actively breastfeeding (46).

Finally, the ubiquitous reliance on nutritional supplements highlights a fundamental limitation of current management. While deficiencies in iron (as evidenced by low ferritin), vitamin D, and vitamin B12 are common in postpartum women and are known to exacerbate telogen effluvium, supplementation will only be effective if a true deficiency exists (33, 48). For women with normal levels, taking supplements provides no benefit in preventing or treating hair loss. Furthermore, the evidence supporting universal supplementation is weak; many studies are small, not placebo-controlled, or show only marginal benefits compared to a robust placebo effect, as seen with drinkable nutraceuticals (42, 49). Over-supplementation, especially with biotin, is discouraged as it can interfere with laboratory tests such as troponin, leading to false results. Therefore, the current medical arsenal for postpartum telogen effluvium is a combination of partially effective, slow-acting, or potentially harmful options. This clear therapeutic gap underscores a critical and urgent need to develop safe, targeted, and multimechanistic therapies that can effectively address the complex interplay of hormonal, inflammatory, and oxidative stress pathways driving this common condition.

## 4 Molecular mechanisms of UCB-EVs in promoting hair follicle regeneration

### 4.1 Activation of the Wnt/β-catenin signaling pathway

The Wnt/β-catenin signaling pathway is a central regulator of hair follicle development, cycling, and regeneration. Its activation is crucial for the transition from telogen to anagen and for maintaining the proliferation and differentiation of hair follicle stem cells and dermal papilla cells (DPCs). Wnt/β-catenin upregulation by UCB-EVs represents an experimentally validated core regenerative mechanism across PPA-relevant *in vitro* DPC models and postpartum mouse studies (direct functional validation). UCB-EVs (EVs) have emerged as potent modulators of this pathway, primarily due to their cargo of specific proteins and microRNAs that can directly engage the Wnt signaling cascade. For instance, EVs derived from various sources, including those primed with bioactive compounds like curcumin (milk-derived EVs), have been shown to activate the Wnt/β-catenin signaling pathway, remodeling the hair follicle microenvironment and promoting hair regeneration in models of androgenetic alopecia (AGA) (50). This activation involves the stabilization and nuclear translocation of β-catenin, a key transcriptional co-activator. In DPCs, the primary signaling center of the hair follicle, Wnt3a protein and miR-21-5p, delivered via umbilical cord blood EVs, are hypothesized to directly bind to Frizzled receptors and LRP5/6 co-receptors, initiating the canonical cascade. This leads to the phosphorylation and inactivation of GSK-3β within the destruction complex, preventing β-catenin degradation. Consequently, β-catenin accumulates in the cytoplasm and translocates to the nucleus, where it forms a complex with TCF/LEF transcription factors. This complex then upregulates the expression of downstream target genes, including lymphoid enhancer-binding factor 1 (Lef1) and Cyclin D1, which are essential for DPC proliferation and the capacity of these cells to secrete paracrine factors that drive hair follicle growth. Specifically, activated DPCs release key hair-inducing factors such as fibroblast growth factor 7 (FGF7) and bone morphogenetic protein 4 (BMP4), which in turn stimulate the adjacent hair follicle epithelial cells, specifically matrix cells and stem cells, to proliferate and differentiate, thereby promoting active hair shaft formation. Experimental evidence directly supports the following: UCB-EV treatment increases β-catenin protein levels (~2.5-fold) in human dermal papilla cells (DPCs), decreases SFRP1 expression, upregulates Axin2 in mouse hair follicles, and promotes the telogen-to-anagen transition. In contrast, the proposed binding of EV-associated Wnt3a or EV-carried miR-21-5p to Frizzled/LRP5/6 receptors remains hypothetical in the UCB-EV system and has not yet been directly validated. Thus, in **Figure 2**, the pathway “UCB-EVs → β-catenin accumulation → Axin2/Lef1/Cyclin D1 upregulation” should be depicted as experimentally validated, whereas “Wnt3a/miR-21-5p → receptor binding” should be depicted as proposed (2). Summary of UCB-EV studies for hair regeneration with categorized evidence levels are illustrated in **Table 1**.

**Figure 2:**
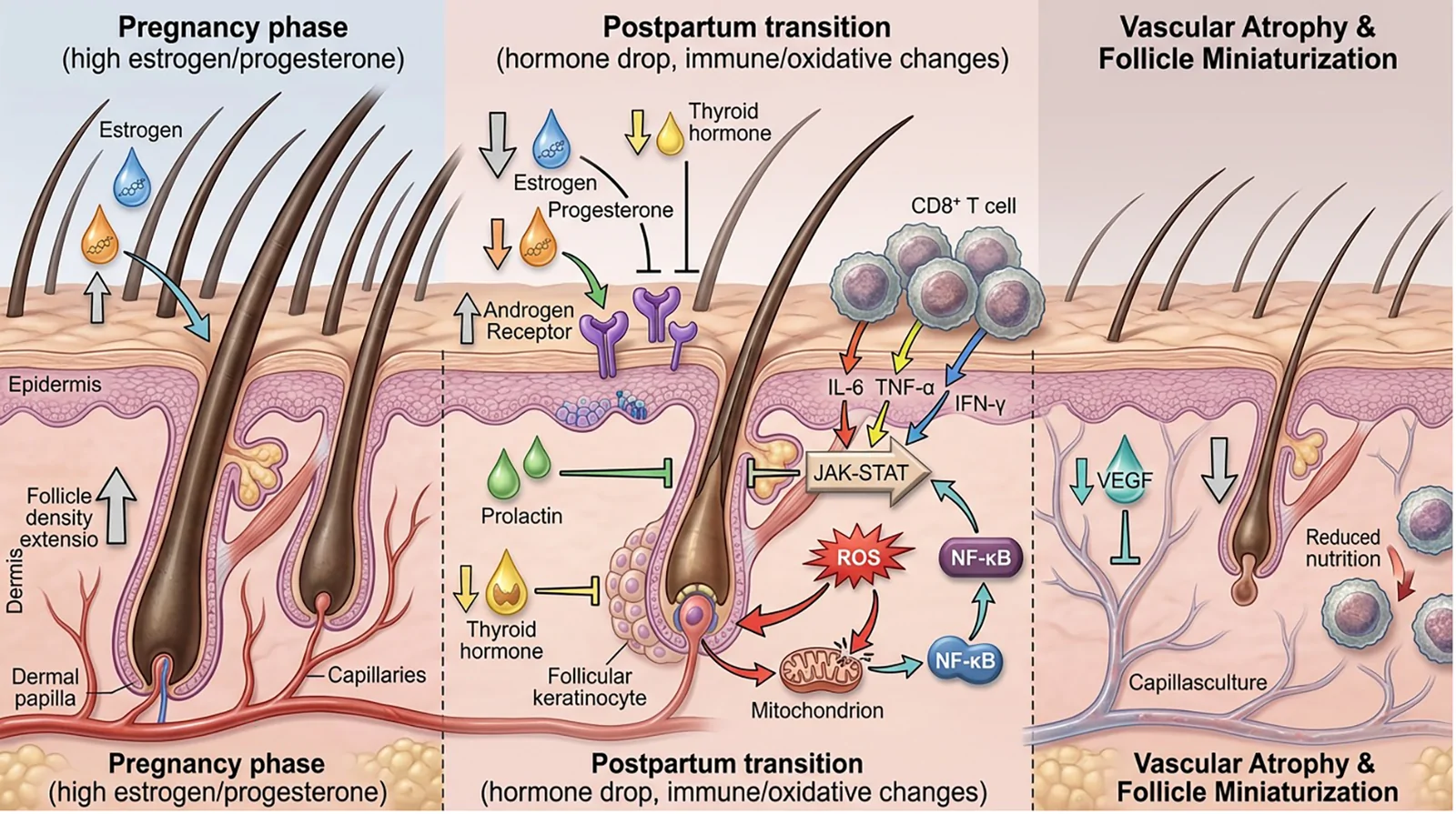
The pathogenesis of postpartum hair loss in women. Solid lines/boxes indicate core PPA pathophysiological pathways (hormonal withdrawal, inflammation/oxidative stress, microvascular impairment); dashed lines/boxes indicate associated or aggravating factors (AGA, TA, thyroid dysfunction, nutritional deficiency).

**Table 1:** Summary of UCB-EV studies for hair regeneration with categorized evidence levels.

EV source	Model	Intervention main mechanism/outcome	Evidence level
UCB-EVs	Human DPC/HFK coculture	50 μg/mL for 48 h	Proliferation ↑45%, apoptosis ↓35%, Versican/ALP ↑,Wnt/β- catenin activation Direct (*in vitro*, PPA-related cells)
UCB-EVs	Cortisol/DHT-induced telogen cell model	*In vitro* treatment	Hair shaft elongation ↑ 30%, restoration of Ki67/PCNA Direct (*in vitro*, hormonal/stress-induced telogen)
UCB-EVs	C57BL/6 PPA mouse model	Intradermal injection every 3 days ×4	Hair coverage ≈80% vs 30%, hair density ↑2-fold, follicle number ↑50%, anagen 20%→65%, CD31 ↑ Direct (*in vivo*, PPA model)
UCB-EVs	vs 2% minoxidil Mouse model	Local injection Comparable hair coverage at day 14; at day 21, anagen 60% (UCB-EVs) vs 40% (minoxidil) Direct comparative (model type to be specified)	hUCMSC-derived exosomes AGA animal model Local administration Rescue from DHT damage, enlarged follicles, telogen→anagen, Wnt/β-catenin activation Indirect (other EV source, AGA model) Macrophage-derived EVs Balb/c mice Injection Wnt3a/7b activation, DPC proliferation/migration ↑, VEGF/KGF ↑ Indirect (other EV source, generic hair growth) Fibroblast-derived EVs Ex vivo human hair follicles In vitro culture Hair shaft elongation, Wnt/βcatenin activation Indirect (other EV source, follicle culture)
UCB-MSC-EVs	Mouse wound healing model	Local treatment	Angiogenesis, ECM remodeling Indirect (wound healing model)

Direct evidence indicates that the intervention was UCB-EV and the model was PPA or PPA-related; indirect evidence indicates other EV sources or non-PPA models. All quantitative values should be verified against the original studies and cited accordingly; if they derive from unpublished work, they should be explicitly labeled as such. elevated measured value = increase.

*In vitro* experiments provide robust evidence for this mechanism. Studies using human hair follicle DPCs treated with umbilical cord blood EVs have demonstrated a significant, approximately 2.5-fold increase in the protein levels of β-catenin, confirming the activation of the pathway (51). Concurrently, the expression of Wnt antagonists, such as secreted frizzled-related protein 1 (SFRP1), is notably decreased, indicating a multi-faceted regulatory effect that removes endogenous brakes on Wnt signaling. The functional consequence of this activation is clearly observed in co-culture models. When DPCs pre-treated with umbilical cord blood EVs are co-cultured with hair follicle epithelial cells, there is a pronounced enhancement of epithelial cell proliferation and the formation of hair shaft-like structures. This demonstrates that EV-mediated signaling is transmitted from the DPCs to the epithelial compartment, effectively recreating the inductive dialogue essential for hair regeneration. The activation of the Wnt/β-catenin pathway is not merely an associative finding but is mechanistically linked to the therapeutic outcomes, as seen in other EV-based therapies where the remodeling of the hair follicle microenvironment through this pathway is a key regenerative event (50).

The *in vivo* significance of this pathway activation is substantiated by animal model studies. In mouse models, subcutaneous injection of umbilical cord blood EVs in the perifollicular region has been shown to accelerate the telogen-to-anagen transition, a critical step in reversing hair loss. Treated mice exhibit a 40% increase in hair follicle length, a direct indicator of enhanced anagen phase duration and hair fiber growth. Furthermore, the expression of the Wnt target gene Axin2, a sensitive reporter of canonical Wnt activity, is significantly upregulated in the hair follicles of EV-treated mice, providing direct *in vivo* molecular evidence for pathway engagement (52). These collective findings collectively demonstrate that umbilical cord blood EVs promote hair follicle regeneration through a Wnt-dependent mechanism, directly targeting the core signaling pathway that governs hair cycling. The ability to mimic the natural inductive signals present in embryonic development and early post-natal life establishes umbilical cord blood EVs as a highly effective biological agent for reactivating dormant DPCs and reversing the miniaturization and cycle arrest characteristic of postpartum hair loss.

### 4.2 Anti-inflammatory and antioxidant stress effects

As described in Section 2.2, inflammation and oxidative stress impair the hair follicle microenvironment through NF-κB/JAK-STAT signaling and ROS/Nrf2 imbalance, respectively. UCB-EVs counteract these processes through several complementary actions: (i) direct reduction of IL-6/TNF-α and CD8+ T-cell infiltration (validated in a PPA mouse model); (ii) delivery of antioxidant enzymes (SOD, CAT, GPX) and activation of the Nrf2 pathway via miR-21 (partially validated *in vitro*); and (iii) induction of regulatory T cells through IL-10/TGF-β1 (proposed, requiring further validation). The following paragraphs detail the evidence level for each mechanism. UCB-EVs are enriched in miR-146a-5p, and studies in other cell/EV systems have shown that this miRNA suppresses NF-κB signaling by targeting TRAF6 and IRAK1. However, direct molecular validation of this axis in the UCBEV/PPA context is still lacking. What has been demonstrated directly is that UCB-EV treatment reduces IL-6 and TNF-α levels and decreases CD8+ T-cell infiltration in PPA mouse models. The miR-146a-5p/TRAF6/IRAK1/NF-κB cascade should therefore be considered a proposed mechanism, while the reduction of inflammatory cytokines and CD8+ T cells can be presented as validated.

Beyond modulating inflammatory cytokine production, umbilical cord blood EVs also directly combat oxidative stress, which is a major driver of DPC senescence and hair follicle dystrophy. The source of EVs, umbilical cord blood, is naturally rich in antioxidant enzymes, and these are packaged into the EVs during their formation. Specifically, umbilical cord blood EVs carry superoxide dismutase (SOD), catalase (CAT), and glutathione peroxidase (GPX), which provide a direct enzymatic defense against reactive oxygen species (ROS). Furthermore, EVs contain miR-21, which can post-transcriptionally regulate the nuclear factor erythroid 2-related factor 2 (Nrf2)/antioxidant response element (ARE) pathway. Nrf2 is a master transcription factor that controls the expression of a battery of endogenous antioxidant and cytoprotective genes, including heme oxygenase-1 (HO-1) and NAD(P)H quinone oxidoreductase 1 (NQO1). By activating the Nrf2 pathway, miR-21 enhances the intrinsic antioxidant capacity of DPCs, providing a sustained and self-amplifying defense against oxidative injury. The functional impact of this combined antioxidant strategy is substantial. *In vitro* experiments using hydrogen peroxide (H_2_O_2_) to induce oxidative stress in DPCs have shown that pre-treatment with umbilical cord blood EVs reduces the rate of apoptosis by 50% and decreases intracellular ROS levels by a remarkable 70% (53). This robust protection preserves the viability and function of DPCs, which are highly sensitive to oxidative damage.

The anti-inflammatory effects are further amplified by additional immunomodulatory components within the EVs. Umbilical cord blood EVs carry cytokines such as interleukin-10 (IL-10) and transforming growth factor-beta 1 (TGF-β1). These immunoregulatory molecules can induce the differentiation of naïve T cells into regulatory T cells (Tregs) within the local tissue microenvironment. Tregs are critical for establishing and maintaining immune tolerance, as they actively suppress the activity of effector T cells and other pro-inflammatory immune cells. By promoting Treg differentiation, umbilical cord blood EVs help to create a tolerogenic and anti-inflammatory milieu around the hair follicle, further shielding it from immune-mediated attack. This multi-tiered approach—combining direct enzymatic ROS scavenging, Nrf2 pathway activation, NF-κB pathway inhibition via miR-146a-5p, and Treg induction through IL-10/TGF-β1—provides a comprehensive and highly effective strategy for resolving the inflammation and oxidative stress that characterize postpartum hair loss. This distinct advantage allows umbilical cord blood EVs to not only stop the progression of hair loss but also to create a permissive environment for the subsequent induction of anagen and robust hair regrowth.

### 4.3 Promoting angiogenesis and nutrient supply to hair follicles

Postpartum hair loss is accompanied by perifollicular capillary atrophy (Section 2.2). UCBEVs promote angiogenesis by delivering pro-angiogenic growth factors (VEGF, HGF, bFGF) and modulating microRNAs (miR-210, miR-126). Direct evidence includes enhanced tube formation in HUVECs, increased KDR phosphorylation, and a 2.8-fold increase in perifollicular capillary density in a PPA mouse model. The specific miRNA-target pathways (miR-210/Ephrin A3, miR-126/SPRED1) are supported by studies in other systems and should be considered proposed mechanisms. UCB-EVs contain VEGF, HGF, bFGF, miR-210, and miR-126. Direct evidence in UCB-EV-treated HUVECs and PPA mouse models includes increased tube formation, elevated KDR phosphorylation, increased perifollicular CD31+ capillary density, and enhanced local blood flow. However, the specific contributions of miR210 via Ephrin A3 and miR-126 via SPRED1 have mainly been established in other experimental systems; in the UCB-EV context, these should be viewed as possible contributing mechanisms, not confirmed ones.

The *in vivo* consequences of these pro-angiogenic effects are striking in preclinical models of hair loss. In a postpartum hair loss mouse model, local injection of umbilical cord blood EVs leads to a rapid and robust increase in perifollicular capillary density. By day 7 post-treatment, the number of functional blood vessels surrounding the hair follicles increases by 2.8-fold compared to controls. This dense neovascularization translates directly into improved tissue perfusion, with a measured 50% increase in local blood flow (54). The enhanced blood supply not only provides the hair bulb cells with the oxygen and glucose required for high-rate mitosis but also ensures efficient delivery of the EVs themselves and their bioactive payloads, creating a positive feedback loop for hair regeneration. Moreover, the hypoxic environment that often precedes angiogenesis is also addressed. Treatment with umbilical cord blood EVs upregulates the expression of hypoxia-inducible factor 1-alpha (HIF-1α) in dermal papilla cells (DPCs). HIF-1α is a master transcription factor that allows cells to adapt to low oxygen conditions by promoting glycolysis and stimulating the expression of pro-angiogenic factors like VEGF. This adaptive response helps maintain DPC viability and function under the metabolically demanding conditions of anagen. The restored blood flow likely mitigates the intense hypoxia at the hair bulb, further stabilizing the anagen phase.

The molecular mechanism behind this robust angiogenic effect is further refined by specific microRNAs (miRNAs) carried within the EVs. For instance, miR-210, known as a “hypoxamiR,” is enriched in umbilical cord blood EVs. By targeting and suppressing the expression of Ephrin A3, a negative regulator of angiogenesis, miR-210 enhances endothelial cell survival and capillary formation. Concurrently, miR-126, a frequently studied proangiogenic miRNA, is also present. miR-126 positively regulates VEGF signaling by targeting SPRED1, an intracellular inhibitor of the Ras/MAPK pathway downstream of VEGF receptor activation. By suppressing SPRED1, miR-126 amplifies the angiogenic signal initiated by VEGF, ensuring a more efficient and sustained response. This synergistic action of multiple growth factors (VEGF, HGF, bFGF) and specific miRNAs (miR-210, miR-126) from the EVs works in concert to reconstruct the perifollicular microvascular network from the ground up. This comprehensive revascularization is a cornerstone of the regenerative therapy provided by umbilical cord blood EVs, as it re-establishes the critical supply line of nutrients and oxygen that is essential for sustaining the rapid cell proliferation of the anagen hair follicle and reversing the atrophic changes seen in postpartum hair loss.

## 5 Preclinical evidence for UCB-EVs in treating postpartum hair loss

### 5.1 *In vitro* cell model studies

Before presenting the preclinical data, it is important to establish the level of evidence. Direct evidence refers to results obtained with UCB-EVs (or explicitly named UCB-MSC-EVs) in PPA-relevant models (e.g., postpartum hormonal-withdrawal models, cortisol/DHT-induced telogen cell models, or PPA mouse models). Indirect evidence refers to findings obtained with EVs from other sources (e.g., MSC-EVs, macrophage-derived EVs, fibroblast-derived EVs) or from non-PPA models (e.g., androgenetic alopecia, wound healing). Proposed mechanisms are those inferred from other EV systems or other disease models but not yet experimentally verified in the UCB-EV/PPA context. We use this classification throughout to avoid over extrapolation.

*In vitro* studies utilizing co-culture systems of human dermal papilla cells (DPCs) and hair follicle keratinocytes (HFKs) have been instrumental in establishing the direct pro-regenerative effects of UCB-EVs. When DPCs, the master regulators of hair follicle cycling, were treated with UCB-EVs at a concentration of 50 μg/mL for 48 hours, significant cellular responses were observed, including a 45% increase in proliferation and a 35% reduction in apoptosis (55). This was accompanied by the upregulation of key hair-inductive genes, such as Versican and alkaline phosphatase (ALP), which are critical markers for maintaining the DPC’s ability to induce new hair follicle formation. These findings align with studies on mesenchymal stem cell-derived EVs (MSC-EVs), which have been shown to promote the proliferation of DPCs and outer root sheath (ORS) cells by translocating β-catenin into the nucleus and increasing the expression of target transcription factors like Axin2 and LEF1 (56). Furthermore, the presence of Wnt3a on the surface of fibroblast-derived EVs has been demonstrated to activate the Wnt/β-catenin pathway in DPCs, leading to enhanced hair shaft elongation in cultured human hair follicles (57). In more complex hormone-induced telogen models, where hair cycle arrest is simulated by the addition of high concentrations of cortisol or dihydrotestosterone (DHT), UCB-EVs have shown a remarkable ability to partially reverse this stagnation. Treatment with UCB-EVs under these conditions promoted hair shaft elongation by 30% and restored the expression of anagen-phase markers such as Ki67 and PCNA, indicating a re-entry into the active growth phase (58). This is consistent with the role of EVs in protecting cells from injury, as seen with miR-124-3p-loaded small EVs protecting dopaminergic neurons from 6-hydroxydopamine-induced toxicity (23) and with MSC-EVs mitigating hypoxia/reoxygenation injury in renal cells (59).

Transcriptomic analyses have further elucidated the molecular mechanisms underpinning the regenerative capacity of UCB-EVs by demonstrating their ability to modulate multiple signaling cascades. UCB-EV treatment consistently upregulates genes associated with progrowth pathways such as Wnt, Hedgehog, and FGF, while simultaneously suppressing proapoptotic signals like TGF-β and BMP. This dual action is crucial for shifting the balance from a resting or catagen state toward a proliferative anagen state. The activation of the Wnt/βcatenin pathway is particularly well-documented as a central driver of hair follicle regeneration, a mechanism also leveraged by EVs from other sources. For instance, macrophage-derived EVs enriched with Wnt3a and Wnt7b can activate this pathway in DPCs, enhancing their proliferation, migration, and expression of growth factors like VEGF and KGF (60). Similarly, engineered EVs from hair follicle stem cells have been shown to harness this pathway to fuel hair regrowth (61). In contrast, the ability of UCB-EVs to inhibit TGF-β and BMP signaling is a key advantage, as these pathways are often associated with fibrosis and the promotion of catagen. This comprehensive regulation of signaling networks, which involves the transfer of bioactive molecules such as microRNAs and proteins, provides a robust mechanistic framework for the observed enhancement of DPC and HFK function, confirming the potent biological activity of UCB-EVs at the cellular level.

### 5.2 Animal model studies

The therapeutic potential of UCB-EVs has been rigorously validated *in vivo* using a C57BL/6 mouse model of postpartum hair loss, which mimics the hormonal fluctuations characteristic of the human condition. In this model, mice received intradermal injections of UCB-EVs every three days for a total of four treatments. By day 14 post-initial treatment, the UCB-EV group demonstrated a significantly higher hair growth coverage rate (approximately 80%) compared to the control group (approximately 30%), with a corresponding 2-fold increase in hair density (62). This robust induction of hair growth is consistent with findings from studies utilizing other types of EVs. For example, macrophage-derived EVs injected into Balb/c mice promoted hair follicle growth *in vivo* (60), and apoptotic extracellular vesicles (apoEVs) were shown to promote hair growth by activating the Wnt/β-catenin pathway in skin and hair follicle mesenchymal stem cells (63). Histological analysis of the treated skin provided deeper insights, revealing a 50% increase in the number of hair follicles per microscopic field. More strikingly, the proportion of follicles in the anagen (growth) phase increased from 20% in the control group to 65% in the UCB-EV group, with a corresponding decrease in the telogen (resting) phase (2). This shift in the hair cycle is a critical indicator of successful therapeutic intervention. Furthermore, the UCB-EV treated group showed a significant increase in the area positive for CD31, a marker for endothelial cells, confirming that UCB-EVs promote angiogenesis, a process vital for supplying nutrients and oxygen to the growing follicle. This pro-angiogenic effect aligns with findings from other studies, such as those showing that UCBMNC-sEVs promote angiogenesis and extracellular matrix remodeling in a mouse model of delayed wound healing (13) and that EVs from hair follicle-derived MSCs can increase angiogenesis in human umbilical vein endothelial cells (64).

A critical aspect for the clinical translation of any new therapeutic is its safety profile. The same animal studies demonstrated that local injection of UCB-EVs did not induce any adverse effects such as local redness, swelling, or inflammation. Comprehensive safety assessments showed that serum biochemical markers for liver and kidney function, as well as complete blood counts, remained within normal ranges. Immunohistochemical analysis further revealed no abnormal infiltration of CD3+ T cells or F4/80+ macrophages at the injection sites, indicating that the treatment did not elicit a significant immune response. This favorable safety profile is a notable advantage and is consistent with the broader literature on the toxicological profile of UCB-derived small EVs. A dedicated study on UCB-MNC-sEVs found no significant toxicity *in vitro* or *in vivo*, even after repeated high-dose intravenous administration, with no changes in hematological parameters or morphological alterations in major organs (17). Furthermore, MSC-derived EVs are generally considered safe and have been used in various models, including for systemic treatment of conditions like diabetic kidney disease (65) and psoriasis (66), without major adverse events. The lack of immunogenicity is likely due to the inherent prope01rties of umbilical cord blood components, which are known for their low immunogenicity (67) and their ability to modulate T cell responses, as seen with cord blood plasma-derived EVs that inhibit T cell proliferation by modulating IL-2 signaling (20). Collectively, these preclinical results provide strong evidence for both the efficacy and the excellent biocompatibility of UCB-EVs, supporting their candidacy as a safe and potent cell-free therapy for postpartum hair loss.

### 5.3 Comparative studies with existing treatments

To contextualize the therapeutic value of UCB-EVs, direct comparative studies with established treatments like minoxidil have been performed in postpartum telogen effluvium mouse models (direct PPA-specific evidence). In a head-to-head comparison using a mouse model, both UCB-EVs and 2% minoxidil solution achieved comparable initial rates of hair regeneration, with no significant difference in hair coverage by day 14. However, a key difference emerged concerning the durability of the effect. The UCB-EV-treated group maintained a significantly longer anagen phase, with 60% of follicles still in the growth phase at day 21, whereas the minoxidil group had already transitioned a substantial number of follicles into catagen/telogen, with only 40% remaining in anagen. This suggests that while minoxidil effectively triggers the initial onset of anagen, UCB-EVs may provide a more sustained regenerative signal, potentially by better modulating the underlying signaling pathways of the hair follicle cycle (68). This sustained benefit is a crucial advantage, as it could translate to less frequent dosing and more robust long-term hair growth in a clinical setting. Mechanistically, the ability of UCB-EVs to provide a complex cocktail of bioactive molecules—including growth factors, cytokines, and microRNAs—that simultaneously activate multiple pro-growth pathways (e.g., Wnt, Hedgehog) while inhibiting pro-apoptotic ones (e.g., TGF-β, BMP) may explain this more durable effect compared to the single-agent action of minoxidil.

Compared to platelet-rich plasma (PRP), a widely used regenerative therapy, UCB-EVs offer several practical and biological advantages. From a manufacturing perspective, UCB-EVs can be produced as an” off-the-shelf, “standardized product, whereas PRP is autologous, leading to significant batch-to-batch variability dictated by the patient’s own blood composition. This standardization and the absence of cellular components eliminate the risk of cellular contamination or immune rejection. Biologically, in cell proliferation assays using DPCs, UCB-EVs showed a 1.5-fold higher efficiency in promoting proliferation compared to PRP (69). Furthermore, UCB-EVs exhibited stronger anti-inflammatory activity, as evidenced by a higher inhibition rate of the pro-inflammatory cytokine IL-6. This superior antiinflammatory profile is consistent with the well-documented immunomodulatory capabilities of cord blood-derived products, such as the conditioned media from umbilical cord-derived MSCs, which can promote anti-inflammatory macrophage polarization despite a proinflammatory cytokine profile (70). The ability to suppress inflammation is particularly relevant for postpartum hair loss, which can be exacerbated by inflammatory processes. Additionally, the exploration of combination therapies has highlighted the potential synergistic effects of UCB-EVs. For instance, combining UCB-EVs with low-level laser therapy (LLLT) resulted in a 60% greater increase in hair follicle length compared to either treatment alone, along with stronger activation of the Wnt signaling pathway (14). This synergistic potential has been observed with other EV-based approaches, such as the co-delivery of finasteride and gold nanoparticles within hybrid hair follicle stem cell EVs, which enhanced treatment outcomes in a model of androgenetic alopecia (61). These comparative and combinational data suggest that UCB-EVs are not only effective as a stand-alone therapy but also hold great promise as an adjunctive agent to augment the efficacy of existing treatments, potentially leading to more effective and personalized treatment protocols for hair loss.

## 6 Clinical translation challenges and future directions of UCB-EVs for postpartum hair loss

### 6.1 Large-scale production and quality control

The successful clinical translation of umbilical cord blood (UCB)-derived extracellular vesicles (EVs) for postpartum alopecia requires the establishment of robust and scalable manufacturing processes coupled with stringent quality control (QC) measures, with unresolved translational barriers including donor heterogeneity, cargo variability, lack of universal hair-specific potency assays, and region-specific cell therapy regulatory frameworks. Donor and cargo heterogeneity barriers: UCB-EV yield, miRNA/growth factor cargo composition vary drastically based on maternal gestational health, gestational age, labor complications, and cord blood collection timing; no universal donor exclusion/inclusion criteria exist to standardize vesicle regenerative potency. Even identical isolation protocols produce heterogeneous EV subpopulations with divergent anti-inflammatory vs pro-angiogenic cargo profiles, creating inconsistent *in vivo* hair regenerative outcomes across batches. A primary hurdle is the limited volume of UCB obtainable per unit, as a single cord blood donation yields only approximately 10^10 to 10^11 EV particles. To meet the substantial dose requirements for therapeutic applications (e.g., 5×10^10 particles for an adult human scalp), the development of large-scale culture systems is essential. A pivotal strategy involves expanding UCB-derived mesenchymal stem cells (UCBMSCs) in bioreactors, which can dramatically amplify EV production. For instance, the use of a chemically defined, xeno- and blood-free culture medium, such as Oxium™EXO, has been shown to increase small EV (sEV) secretion from MSCs by three-fold, consistently across multiple harvest time points and different cell sources, yielding an enrichment of particles within the 51–200 nm range (71). Furthermore, the optimization of isolation techniques is critical; replacing traditional ultracentrifugation (UC) with a combination of ultrafiltration and size exclusion chromatography (UF/SEC) can recover approximately 400 times more sEV per milliliter of conditioned medium, and this process is inherently more scalable and standardized (13). This approach not only enhances yield but also minimizes downstream contaminants while preserving the vesicles’ regenerative properties, such as their capacity to promote angiogenesis and extracellular matrix remodeling (13).

Parallel to production scalability, establishing a rigorous QC framework is paramount to ensure batch-to-batch consistency and clinical safety. The QC system must evaluate the identity, purity, potency, and safety of each EV batch. For identity and purity, following the guidelines of the International Society for Extracellular Vesicles (ISEV), specifications for particle size distribution (e.g., via nanoparticle tracking analysis), concentration, and the presence of specific surface markers (e.g., CD63, CD9, CD81) are mandatory (72). The absence of cellular contaminants and the confirmation of sterility, mycoplasma, and endotoxin levels are also vital. Potency assay limitations: No globally harmonized, PPA-specific functional potency assay is currently accepted by regulatory bodies. Current surrogate tests (*in vitro* DPC proliferation, miRNA quantification) cannot fully recapitulate *in vivo* scalp inflammatory/oxidative microenvironment responses required for clinical efficacy prediction. For postpartum alopecia, a relevant potency test could be an *in vitro* hair dermal papilla cell (DPC) proliferation assay or the quantitative profiling of key miRNAs (e.g., miR-21, miR146a) that are known to be involved in hair follicle cycling and inflammation (13). This approach was successfully adopted in studies where UCB-MSC-derived EVs were manufactured in compliance with Korean MFDS guidelines; the final product was characterized for basic traits (size, zeta potential, marker expression) and its functional efficacy was validated through *in vitro* cell proliferation and migration assays, as well as *in vivo* animal models (72). Regulatory barriers: Classified as cell-derived biological products in most jurisdictions, UCB-EVs require full IND/biologics license application pathways with long-term toxicology, reproductive safety (critical for breastfeeding postpartum populations), and batch comparability data; harmonized global GMP standards for cord blood EV manufacture for dermatological indications remain absent.

Despite the immense promise of exosome-based therapies, their clinical translation is often hindered by a limited yield and variability in potency, particularly when derived from primary cells such as those from umbilical cord blood. A major challenge lies in obtaining sufficient quantities of functional exosomes for therapeutic application. To overcome this bottleneck, a recent and innovative strategy involves the pretreatment of parental cells to enhance the efficacy of their secreted exosomes (73). Among various approaches, melatonin pretreatment has emerged as a particularly effective method. Melatonin, a potent antioxidant and antiinflammatory agent, has been shown to significantly enhance the therapeutic potential of exosomes by enriching their cargo with protective miRNAs and proteins, thereby amplifying their anti-inflammatory, antioxidant, and anti-apoptotic functions (73, 74). This strategy has demonstrated notable benefits in ameliorating ischemia-reperfusion injury, chronic kidney disease, and diabetic wound healing by modulating pathways such as PTEN/AKT, TLR4/NFκB, and Sirt signaling (73, 74). However, this approach has not yet been explored in the context of alopecia. Given the established roles of oxidative stress, inflammation, and regulated cell death in hair follicle pathology, we hypothesize that melatonin pretreatment could serve as a novel strategy to boost the therapeutic efficacy of human umbilical cord blood-derived exosomes for alopecia treatment. Future studies are warranted to evaluate whether melatonin preconditioned umbilical cord blood exosomes exhibit superior hair regenerative capacity.

Finally, the long-term storage stability of UCB-EVs is a critical consideration for clinical logistics and distribution. EVs are biologically labile and prone to degradation or aggregation during storage. Lyophilization (freeze-drying), particularly with the addition of cryoprotectants like trehalose, has emerged as a promising strategy to extend the shelf life of UCB-EVs to over six months while retaining more than 80% of their biological activity. This approach is supported by studies on other MSC-derived EVs, where functional properties such as internalization into target cells and systemic biodistribution *in vivo* were preserved after optimized storage (74). The choice of storage buffer, temperature (e.g., -80 °C for liquid storage vs. 4 °C for lyophilized product), and container material all require meticulous optimization. Advanced formulations, such as combining EVs with biocompatible polymers or embedding them in hydrogels, could further enhance stability and facilitate “ready-to-use” clinical products. In summary, the path from laboratory discovery to clinical application depends on overcoming the dual challenges of large-scale production and harmonized quality control, using methods that are not only efficient but also compliant with Good Manufacturing Practice (GMP) standards.

Beyond the manufacturing process, donor variability and cargo heterogeneity are major determinants of batch consistency. The yield and molecular composition of UCB-EVs are influenced by maternal age, gestational age, mode of delivery, cord blood collection and transport time, and donor health status; thus, miRNA and protein profiles may differ markedly between batches. Particle size and tetraspanin markers alone are therefore insufficient for quality control. We propose a set of critical quality attributes (CQAs) for each batch: (1) particle concentration and size distribution by nanoparticle tracking analysis; (2) EV markers (CD63/CD81/TSG101) and negative markers (e.g., calnexin/APOB); (3) key functional molecular fingerprints (e.g., miR-21, miR-146a, VEGF); (4) biological potency assays (e.g., dermal papilla cell proliferation, HUVEC tube formation, or anti-inflammatory activity); and (5) bioburden, sterility, mycoplasma, and endotoxin testing. From a regulatory standpoint, UCB-EVs are biological products with no globally harmonized approval framework; FDA, EMA, and NMPA currently classify them as drug products or advanced therapy medicinal products (ATMPs), requiring GMP-compliant CMC documentation, non-clinical toxicology, and phased clinical trials. Early regulatory consultation is strongly advised.

### 6.2 Route of administration and dose optimization

The clinical efficacy of UCB-derived EVs for postpartum alopecia is highly dependent on the route of administration and the optimized dose, as these factors directly influence EV bioavailability, targeting, and overall therapeutic outcome. Local injection has been the initial preference due to its direct delivery to the scalp, but it is associated with significant drawbacks, including procedural pain, poor patient compliance, and potential for uneven distribution. To mitigate these issues, microneedling has been explored as a physical enhancement strategy. Microneedling creates transient microchannels in the stratum corneum, thereby markedly increasing the transdermal permeation of EVs. Preclinical evidence suggests that combining microneedling with UCB-EV therapy yields superior hair regrowth compared to topical application or injection alone, offering a nearly non-invasive and more patient-friendly alternative.

Despite these advances, topical formulations such as gels, creams, or sprays remain the most desirable administration route for their non-invasiveness, ease of self-application, and high patient acceptability. The primary challenge for topical delivery is the rapid degradation and poor penetration of EVs through the skin barrier. To circumvent this, advanced drug delivery systems are being developed. Encapsulating UCB-EVs within liposomes or loading them into hyaluronic acid (HA)-based hydrogels can provide sustained release profiles, with studies demonstrating a 70% release rate over 48 hours, and can significantly enhance follicular targeting by exploiting the natural affinity of HA for hair follicle structures (75). These biomaterials protect the EVs from enzymatic degradation and facilitate deeper penetration into the dermis where hair follicle stem cells reside. Furthermore, the use of lipid-based carriers can be engineered to actively target specific cell surface receptors, such as those on DPCs or hair follicle stem cells, further improving therapeutic precision (76, 77).

Dose optimization is equally critical, as both under- and over-dosing can lead to suboptimal results or potential adverse effects. Data from murine models of alopecia indicate an effective dose of approximately 10^8 particles per square centimeter of skin. When scaling this to the human scalp, which has an average surface area of about 500 cm², the required dose is estimated to be around 5×10^10 EV particles. However, this is a rough estimate; the actual effective dose may vary based on factors like disease severity, route of administration, and the specific potency of the EV batch. It is imperative to conduct dose-escalation clinical trials to systematically determine the optimal dosage for postpartum alopecia. Such trials should also determine the optimal treatment frequency (78). Based on the known *in vivo* half-life of EVs (typically 6–24 hours after local administration) and the regenerative cycle of the hair follicle, a regimen of 1–2 applications per week for a total treatment course of 8–12 weeks appears logical. Finally, the biodistribution and pharmacokinetics of topically applied EVs are crucial for ensuring safety. Studies in animal models have shown that systemically administered EVs (e.g., intraperitoneal) have a defined biodistribution profile that can be influenced by formulation (71). For topical use, the primary goal is to maximize local retention while minimizing systemic absorption, thereby reducing off-target effects. Ultimately, a multi-faceted approach combining advanced formulation (e.g., hydrogels) with a physical enhancement technique (e.g., microneedling) and a rationally designed dosing schedule will be most effective for this indication.

### 6.3 Safety assessment and individualized therapy

The advancement of UCB-EVs into clinical practice for postpartum alopecia necessitates a comprehensive safety assessment and the development of personalized treatment strategies to maximize efficacy and minimize risks. One of the most attractive features of UCB-EVs is their inherently low immunogenicity. Preclinical safety evaluations using non-human primates have shown that high-dose intravenous administration of human UCB-MSC-derived exosomes (3.85 × 10^12 particles) did not cause significant adverse effects on general clinical conditions, hematology, coagulation, or immune organs, and did not induce major changes in immunoglobulins or cytokines (79). However, longer-term safety assessments are essential before clinical translation. These should include the evaluation of local tissue tolerance (e.g., irritation, allergic reactions), potential systemic toxicity, and the theoretical risk of inducing tumorigenesis. Given that EVs can carry growth factors and signaling molecules, their impact on the scalp’s microbiome and the balance between anagen (growth) and catagen (regression) phases should be rigorously assessed. A full toxicology program, including repeated-dose toxicity studies and genotoxicity testing, should be integrated into the Investigational New Drug (IND) application. Existing safety data are derived mainly from short-term animal studies and non-human primate single/repeat-dose studies; long-term carcinogenicity, immunogenicity (e.g., anti-EV antibody formation), reproductive toxicity, and data on use during lactation are still lacking. For postpartum women, many of whom are breastfeeding, systemic exposure to any exogenous biologic requires particular caution. Accordingly, the wording “safe” should be avoided in favor of “well tolerated in available preclinical studies, with long-term safety still to be established.”

A second major challenge is the inherent heterogeneity among postpartum alopecia patients, which can arise from differences in hormonal profiles (e.g., levels of prolactin, estrogen, and androgens), genetic predisposition (e.g., variations in androgen receptor sensitivity), postpartum stress, and concurrent medical conditions such as thyroid dysfunction or iron deficiency. This variability suggests that a “one-size-fits-all” approach may be suboptimal. Future therapeutic strategies should evolve toward personalized EV-based treatments. For example, a pre-treatment biopsy or a non-invasive scalp tape-stripping could be used to characterize each patient’s scalp microenvironment, focusing on the expression of specific miRNAs (e.g., miR-21, miR-146a) or inflammatory cytokines (e.g., TNF-α, IL-6). Patients with a high-inflammatory subtype would theoretically benefit more from an EV preparation enriched with anti-inflammatory molecules, such as an EV subpopulation engineered or selected for high miR-146a content. This is analogous to the concept of “EV fingerprinting” where the internal cargo (proteins, lipids, nucleic acids) is defined and used for targeted therapy (71). UCB-MSC-EVs are inherently rich in regenerative mediators like hemopexin and miR150 (14), but specific enrichment for a desired therapeutic effect is a promising avenue.

Finally, the future of UCB-EV therapy for postpartum alopecia likely lies in combination strategies. Combining UCB-EVs with other biological materials, such as collagen scaffolds or growth factor cocktails (e.g., VEGF, FGF), can create a more comprehensive regenerative microenvironment. Additionally, integrating UCB-EVs with established pharmacotherapies, such as topical minoxidil or low-dose oral spironolactone, may synergistically enhance hair regrowth by targeting complementary pathways (e.g., growth promotion vs. androgen receptor blockade). To validate the clinical value, rigorous, multi-center, randomized, double-blind, placebo-controlled clinical trials are essential. These trials should not only evaluate hair count and density but also incorporate patient-reported outcome measures (e.g., quality of life, satisfaction) and long-term safety surveillance. A systematic review of stem cell therapies for erectile dysfunction highlights that while the field shows promise, the lack of standardized protocols and controlled groups in many studies hinders trial comparability (80). The translation of UCB-EVs for alopecia must learn from these lessons and adhere to the highest levels of evidence-based clinical research.

## 7 Conclusion

UCB-EVs represent a promising investigational cell-free candidate for future postpartum hair loss intervention based solely on preclinical data, offering a novel, cell-free strategy that serves as a theoretical translational link between regenerative medicine and clinical dermatology; robust human clinical trial evidence confirming clinical efficacy and long-term breastfeeding safety is entirely absent to date. As articulated in the preceding sections, UCB-EVs, enriched in a diverse cargo of microRNAs, growth factors, and anti-inflammatory proteins, exert a multifaceted regenerative effect on hair follicles. Their mechanisms are orchestrated, ranging from the activation of the Wnt/β-catenin signaling pathway—a master regulator of hair follicle cycling—to the suppression of inflammatory cascades and oxidative stress, and the promotion of robust pro-angiogenic activity. This convergence of actions not only reverses the telogen arrest characteristic of postpartum hair loss but also promotes anagen re-entry, thereby increasing hair density and growth velocity.

Recent advances have illuminated the critical role of regulated cell death (RCD), particularly ferroptosis, in hair follicle biology and the pathogenesis of alopecia. Transcriptomic analyses have revealed the involvement of immune cells and ferroptosis in frontal fibrosing alopecia (81). In alopecia areata, multiple ferroptosis-related genes, including SLC40A1, LCN2, CREB5, and SLC7A10, have been identified as potential diagnostic biomarkers, with their expression significantly downregulated in lesions and inversely correlated with disease severity (82). The localization of these genes’ protein products to the outer root sheath and sebaceous glands further suggests a link between metabolic dysregulation and hair loss (82). These findings underscore the potential of targeting RCD pathways for therapeutic intervention.

Concurrently, umbilical cord blood-derived cells and their secreted exosomes have emerged as promising regenerative tools, known for their potent immunomodulatory and anti-inflammatory properties. Notably, recent evidence suggests that umbilical cord blood-derived exosomes may exert a protective effect against ferroptosis (83). This raises the compelling hypothesis that umbilical cord blood cells and their exosomes could ameliorate alopecia by modulating RCD pathways, such as ferroptosis. Future studies are warranted to systematically investigate this hypothesis, exploring whether these perinatal derivatives can protect hair follicle cells from ferroptotic damage, regulate the expression of key ferroptosis-related genes, and ultimately promote hair regeneration.

The most compelling attribute of UCB-EVs is their ability to address the complex pathophysiology of postpartum hair loss. Traditional treatments, such as minoxidil, primarily target stimulating hair growth while failing to adequately mitigate the underlying inflammatory and microvascular changes that occur in the postpartum period. In contrast, UCB-EVs provide a holistic, biomimetic intervention. The pre-clinical evidence, derived from both *in vitro* human dermal papilla cell models and *in vivo* murine models, is consistent and promising. However, a balanced interpretation necessitates recognizing that while these models are essential for mechanistic validation, they do not fully recapitulate the chronic, hormonally dynamic environment of the human postpartum scalp. For instance, the rapid clearance of exogenously administered EVs in small animals may not reflect the sustained bioavailability required for clinical efficacy in humans. Nevertheless, the demonstrated equivalence to or superiority over minoxidil in animal models is a significant milestone, underscoring the therapeutic potential of UCB-EVs.

The development of UCB-EVs as a therapeutic asset, however, is not without its formidable challenges. The field currently stands at a crossroads where the translational hurdles are as significant as the therapeutic promise. The foremost challenge is scalability and standardization. Unlike chemical drugs, UCB-EVs are biological entities whose composition and potency are influenced by maternal health, umbilical cord processing, and isolation methods. Balancing the need for high-yield, cost-effective production with rigorous quality control is a delicate act. From a regulatory perspective, establishing standardized release criteria—such as particle size, miRNA profile, and functional potency—is non-negotiable for reproducible clinical outcomes. This balance demands a collaborative effort between academic researchers and biopharmaceutical industries to develop Good Manufacturing Practice (GMP)compliant protocols.

Another critical dimension is the optimization of delivery. Topical application, while desirable for patient compliance and safety, is hampered by the stratum corneum barrier and rapid degradation of EVs in the follicular environment. The proposed solutions, such as microneedling and biomaterial-based scaffolds, represent a sophisticated response to this challenge. Microneedling, by creating transient microchannels, enhances EV penetration, while biomaterials like hydrogels provide a sustained-release depot. Balancing these approaches requires careful risk-benefit analysis: microneedling must avoid excessive trauma that could trigger an inflammatory rebound, and biomaterials must be biocompatible and fully degradable. The expert perspective here is to adopt a “precision delivery” paradigm, where the carrier is tailored to the individual’s scalp sensitivity and extent of hair loss.

Looking forward, the future of UCB-EVs in postpartum alopecia lies in engineering intelligence. The concept of “next-generation” EVs—modified to carry specific proregenerative miRNAs (e.g., miR-218-5p or miR-146a) or functionalized with hair follicletargeting ligands (e.g., anti-CD133 or vitronectin-mimetic peptides)—is a logical evolution. This technological advancement would allow us to augment their intrinsic therapeutic strength while mitigating variability. However, this raises a crucial balance between nature and engineering. Over-engineering could risk off-target effects or immunogenicity. Therefore, research must prioritize safety over efficacy increments, ensuring that these modifications are clinically validated before widespread adoption.

Ultimately, the most pragmatic path forward involves a tiered strategy. Initially, clinical trials should focus on established, scalable EV products with validated mechanisms. These trials must be designed with rigorous endpoints, including trichoscopic measurement, standardized photography, and patient-reported outcomes. Future studies should then explore combinatorial approaches, such as UCB-EVs plus low-level laser therapy or platelet-rich plasma, to harness synergistic effects while reducing individual therapy burden. For postpartum patients—often breastfeeding mothers—safety is paramount. The cell-free nature of EVs theoretically reduces risks associated with cell-based therapies, but long-term data on immune modulation and off-target differentiation are needed.

In conclusion, UCB-EVs offer a scientifically robust and therapeutically promising avenue for treating postpartum hair loss. The journey from bench to bedside requires us to balance mechanistic rigor with translational pragmatism, to harmonize the promise of nature-derived therapeutics with the precision of synthetic biology, and to prioritize patient-centered safety as we scale up from laboratory innovation to clinical reality. With coordinated efforts in standardization, delivery engineering, and evidence-based trials, UCB-EVs could soon redefine the standard of care for millions of women navigating the distressing aftermath of postpartum hair loss.
